# Systematic reduction of gray matter volume in anorexia nervosa, but relative enlargement with clinical symptoms in the prefrontal and posterior insular cortices: a multicenter neuroimaging study

**DOI:** 10.1038/s41380-023-02378-4

**Published:** 2024-01-22

**Authors:** Keima Tose, Tsunehiko Takamura, Masanori Isobe, Yoshiyuki Hirano, Yasuhiro Sato, Naoki Kodama, Kazufumi Yoshihara, Norihide Maikusa, Yoshiya Moriguchi, Tomomi Noda, Ryo Mishima, Michiko Kawabata, Shun’ichi Noma, Shu Takakura, Motoharu Gondo, Shingo Kakeda, Masatoshi Takahashi, Satoru Ide, Hiroaki Adachi, Sayo Hamatani, Rio Kamashita, Yusuke Sudo, Koji Matsumoto, Michiko Nakazato, Noriko Numata, Yumi Hamamoto, Tomotaka Shoji, Tomohiko Muratsubaki, Motoaki Sugiura, Toshiya Murai, Shin Fukudo, Atsushi Sekiguchi

**Affiliations:** 1 Department of Psychiatry, Graduate School of Medicine, Kyoto University Hospital, Kyoto, Japan; 2 Department of Behavioral Medicine, National Institute of Mental Health, National Center of Neurology and Psychiatry, Tokyo, Japan; 3 Research Center for Child Mental Development, Chiba University, Chiba, Japan; 4 United Graduate School of Child Development, Osaka University, Kanazawa University, Hamamatsu University School of Medicine, Chiba University and University of Fukui, Suita, Japan; 5 Department of Psychosomatic Medicine, Tohoku University Hospital, Sendai, Japan; 6 Division of Psychosomatic Medicine, Department of Neurology, University of Occupational and Environment Health, Kitakyushu, Japan; 7 Department of Psychosomatic Medicine, Kyushu University Hospital, Fukuoka, Japan; 8 Center for Evolutionary Cognitive Sciences, Graduate School of Art and Sciences, The University of Tokyo, Tokyo, Japan; 9 Nomakokoro Clinic, Kyoto, Japan; 10 Department of Radiology, Hirosaki University Graduate School of Medicine, Aomori, Japan; 11 Department of Radiology, University of Occupational and Environmental Health, School of Medicine, Kitakyushu, Japan; 12 Department of Neurology, University of Occupational and Environmental Health School of Medicine, Kitakyushu, Japan; 13 Research Center for Child Mental Development, University of Fukui, Fukui, Japan; 14 Department of Radiology, Chiba University Hospital, Chiba, Japan; 15 Department of Psychiatry, International University of Health and Welfare, School of Medicine, Narita, Japan; 16 Department of Cognitive Behavioral Physiology, Graduate School of Medicine, Chiba University, Chiba, Japan; 17 Department of Psychology, Northumbria University, Newcastle-upon-Tyne, United Kingdom; 18 Department of Human Brain Science, Institute of Development, Aging, and Cancer, Tohoku University, Sendai, Japan; 19 Department of Internal Medicine, Nagamachi Hospital, Sendai, Japan; 20 Department of Psychosomatic Medicine, Tohoku University School of Medicine, Sendai, Japan; 21 Department of Psychosomatic Medicine, Tohoku University Graduate School of Medicine, Sendai, Japan; 22 Cognitive Sciences Lab, International Research Institute of Disaster Science, Tohoku University, Sendai, Japan; 23 Center for Eating Disorder Research and Information, National Center of Neurology and Psychiatry, Tokyo, Japan; 24 Department of Advanced Neuroimaging, Integrative Brain Imaging Center, National Center of Neurology and Psychiatry, Tokyo, Japan

**Keywords:** Neuroscience, Psychology

## Abstract

Although brain morphological abnormalities have been reported in anorexia nervosa (AN), the reliability and reproducibility of previous studies were limited due to insufficient sample sizes, which prevented exploratory analysis of the whole brain as opposed to regions of interest (ROIs). Objective was to identify brain morphological abnormalities in AN and the association with severity of AN by brain structural magnetic resonance imaging (MRI) in a multicenter study, and to conduct exploratory analysis of the whole brain. Here, we conducted a cross-sectional multicenter study using T1-weighted imaging (T1WI) data collected between May 2014 and February 2019 in Japan. We analyzed MRI data from 103 female AN patients (58 anorexia nervosa restricting type [ANR] and 45 anorexia nervosa binge-purging type [ANBP]) and 102 age-matched female healthy controls (HC). MRI data from five centers were preprocessed using the latest harmonization method to correct for intercenter differences. Gray matter volume (GMV) was calculated from T1WI data of all participants. Of the 205 participants, we obtained severity of eating disorder symptom scores from 179 participants, including 87 in the AN group (51 ANR, 36 ANBP) and 92 HC using the Eating Disorder Examination Questionnaire (EDE-Q) 6.0. GMV reduction were observed in the AN brain, including the bilateral cerebellum, middle and posterior cingulate gyrus, supplementary motor cortex, precentral gyrus medial segment, and thalamus. In addition, the orbitofrontal cortex (OFC), ventromedial prefrontal cortex (vmPFC), rostral anterior cingulate cortex (ACC), and posterior insula volumes showed positive correlations with severity of symptoms. This multicenter study was conducted with a large sample size to identify brain morphological abnormalities in AN. The findings provide a better understanding of the pathogenesis of AN and have potential for the development of brain imaging biomarkers of AN. Trial Registration: UMIN000017456. https://center6.umin.ac.jp/cgi-open-bin/icdr/ctr_view.cgi?recptno=R000019303.

## Introduction

Anorexia nervosa (AN) is a psychiatric disorder characterized by concerns about body shape and weight, and abnormal eating behaviors. Diagnostic criteria include restricted energy intake, significant underweight, fear of weight gain, and distorted body image. The homogeneity of AN, with a narrow range of age of onset (early adolescence), stereotyped symptoms and course, and large differences in morbidity between men and women, was thought to be much more promising for detecting biomarkers of AN rather than other psychiatric disorders [1].

Although attempts to elucidate pathological conditions and develop biomarkers using brain imaging studies of AN have been conducted worldwide, the reproducibility and validity of the results remain questionable due to insufficient sample sizes. Neuroimaging findings indicated that gray matter (GM) reduction associated with acute malnutrition in AN was largely reversed by weight regain, at least in younger, non-chronic patients [2, 3]. Associations were detected between differences in brain circuitry of the reward system and different types of eating disorders [4], as well as those related to body image distortion [5, 6], symptoms such as overeating [7], and some cognitive dysfunctions in AN [8–11]. However, these findings were mostly inconsistent, because of small sample sizes and single-center studies. A review of AN structural brain imaging studies showed that most were likely to produce false-positive or false-negative results due to small sample sizes (*n* < 20 patients) and low statistical power [12]. Although, some other meta-analyses indicated decreased GMV in the bilateral cerebellum, middle and posterior cingulate gyrus, precuneus, and supplementary motor cortex [13], most of these studies involved analyses of regions of interest (ROIs) based on hypotheses, founded on previous studies. Therefore, they were prone to publication bias and may have inhibited new discoveries by exploratory analysis (i.e., whole-brain analysis). A search of major clinical research registries, such as ClinicalTrials.gov in the USA and EU Register in the EU, did not identify any multicenter brain imaging studies with regard to AN. Furthermore, no AN brain imaging database is available at present.

In a notable effort, the ENIGMA Eating Disorders Working Group led by Walton et al., aggregated meta-data from 685 females diagnosed with AN and 963 female HC [14]. The ENIGMA project has significantly advanced our understanding of AN, leveraging a meta-analytical approach to aggregate and harmonize data across multiple sites without requiring the sharing of raw data or computational resources [15]. Yet, while providing valuable overarching insights, this approach may overlook specific patterns unique to individual data sets or nuances that emerge from uniform data collection at a single site. Therefore, to ensure a robust and comprehensive understanding of AN’s impact on brain morphology, it remains essential to conduct multicenter studies with a large number of patients. This enables the exploration of whole-brain morphological abnormalities in AN beyond the ROI analyses, while also allowing for the integration and correlation analysis of uniform psychological data.

This study was designed to identify brain morphological abnormalities in AN and their associations with the severity of AN. We accomplished this by conducting a whole-brain voxel-based morphometry (VBM) analysis of structural MRI images collected from patients with AN through a multicenter collaboration. To the best of our knowledge, our study represents the largest VBM study in AN to date, thereby providing a more comprehensive view of the brain abnormalities in AN than previous studies. Our study did not limit the analysis to predetermined ROIs, thus constituting a novel, whole-brain exploration of structural differences in AN. In multicenter MRI studies, it is necessary to correct for intercenter differences in MR images (harmonization). In this study, we integrated and preprocessed data from five centers using the latest harmonization method [16], corrected for intercenter differences, to identify brain morphological changes characteristic of AN.

## Methods

### Eligibility and exclusion criteria

Data from female AN patients who met Diagnostic and Statistical Manual of Mental Disorders, 5th edition (DSM-5) diagnostic criteria, and age-matched healthy females were enrolled for this study. Diagnosis and AN subtype were determined by a semi-structured interview based on the DSM-5 by physicians. HC participants were carefully selected and screened for neuropsychiatric disorders. This was ensured through a semi-structured interview conducted by clinical experts such as a clinical psychologist and a nurse, or a medical interview conducted by a physician, confirming that no participants in our control group had any neuropsychiatric conditions. Subjects with claustrophobia, head trauma, neurological disorders, or substance abuse were excluded from this study. Instead, AN patients were accepted if they had comorbid depression, bipolar disorder, obsessive-compulsive disorder, anxiety disorder, or personality disorder, which are often associated with AN, but were excluded if they had thoughts of imminent death or if these mental disorders were severe enough to warrant hospitalization, and were excluded if they had a history of mental disorders other than the above mental disorders was also excluded from the study. The patients included in our study were individuals who had been expected to be established treatment relationships at their facilities, having provided their consent to participate in this study. As AN is characterized by a strong denial of disease state and refusal of treatment, it would not be feasible nor ethical to include untreated status as a condition for research participation. Consequently, we could not control the timing of treatment initiation, which is dependent on when consent from the patient can be obtained. However, existing literature suggests that changes in brain morphology in AN have a larger effect than other psychiatric diseases [14], which led us to assume that the timing of treatment initiation did not significantly impact our findings.

### MRI and psychological datasets

We constructed the first version of MRI and psychological dataset including 205 right-handed female participants: 103 with AN (58 with anorexia nervosa restricting type [ANR] and 45 with anorexia nervosa binge-purging type [ANBP]) and 102 healthy age-matched HC at five facilities (Table 1). AN participants were diagnosed at each institute according to the DSM-5. All participants provided written informed consent, and this study was approved by the ethics committee of the National Center of Neurology and Psychiatry (A2019-097).

**Table 1:** Participants in Each Center.

Center	AN	{ANR	ANBP}	HC	All
Chiba University	11 (2)	{5 (1)	6 (1)}	19 (10)	30 (12)
Kyushu University	3 (3)	{3 (3)	0 (0)}	4 (4)	7 (7)
Kyoto University	43 (42)	{20 (20)	23 (22)}	50 (49)	93 (91)
Tohoku University	36 (30)	{21 (18)	15 (12)}	21 (21)	57 (51)
University of Occupational and Environmental Health	10 (10)	{9 (9)	1 (1)}	8 (8)	18 (18)
Summary	103 (87)	{58 (51)	45 (36)}	102 (92)	205 (179)

Numbers in parentheses indicate numbers of participants with EDE-Q scores.

*AN* anorexia nervosa, *ANR* anorexia nervosa restricting type, *ANBP* anorexia nervosa binge-purging type, *HC* healthy controls.

Of the 205 participants, severity of eating disorder symptom scores was available for 179 participants, consisting of 87 with AN (51 ANR, 36 ANBP) and 92 HC using the Eating Disorder Examination Questionnaire (EDE-Q) 6.0 [17, 18], a commonly used psychological scale for investigating the severity of eating disorders. EDE-Q is a self-contained, 28-item questionnaire, scored on a 7-point Likert scale (0–6). The global EDE-Q score is the sum of the four subscale scores (Restraint, Eating Concern, Shape Concern, and Weight Concern) divided by 4.

### MRI acquisition parameters

We performed T1-weighted imaging (T1WI) in 3.0-Tesla scanners (GE Discovery MR750 and MR750w; GE Healthcare, Waukesha, WI, USA, Achieva; Philips, Amsterdam, The Netherlands, and MEGNETOM Trio; Siemens Healthcare, Malvern, PA, USA). T1WI data were acquired on 3.0-Tesla scanners. Detailed parameters of the T1WI at each center are summarized in Table 2. For example, whole-brain high-resolution T1-weighted anatomical scans were acquired at Chiba University using a 3D Inversion Recovery Spoiled Gradient Echo (3D IR SPGR) sequence with the following parameters: repetition time (TR) = 7.36 ms, echo time (TE) = 3.05 ms, inversion time (TI) = 400 ms, flip angle = 11°, field of view (FOV) = 256 × 256 mm, acquisition matrix = 256 × 256, slice thickness = 1.0 mm without gap, axial slice number = 196, voxel dimension = 1.00 × 1.00 × 1.00 (see Table 2).

**Table 2:** Scanner types and imaging parameters (T1WI) in each center.

Institute	Chiba University	Kyushu University	Kyoto University	Tohoku University	University of Occupational and Environmental Health
Scanner	GE Discovery MR750 3.0 T	Philips Achieva 3.0 T	Siemens MEGNETOM Trio	Philips Achieva 3.0 T	GE Discovery MR750w 3.0 T
Magnetic field strength (T)	3	3	3	3	3
Number of channels of head coils	32	8	32	8	32
Pulse sequence	3D IR SPGR	MPRAGE	MPRAGE	MPRAGE	3D IR SPGR
Imaging direction	Sagittal	Sagittal	Axial	Sagittal	Sagittal
Matrix	256 × 256	256 × 256	256 × 240	256 × 256	256 × 256
Number of slices	196	200	208	200	196
FOV (mm)	256 × 256	256 × 256	256 × 240	256 × 256	256 × 256
Resolution (mm)	1 × 1	1 × 1	0.9375 × 0.9375	1 × 1	1 × 1
TR (ms)	7.36	7.43	2000	7.28	7.7
TE (ms)	3.05	3.41	3.4	3.38	3.11
TI (ms)	400	900	990	963	400
Slice thickness (mm)	1	1	1	1	1
Flip angle (°)	11	9	8	9	11
Band width (Hz/pixel)	244.141	217	130	217	244.141
Parallel imaging	ASSET 2×	SENSE 2×	No	SENSE 2×	ASSET 2×
Total scan time	4:54	5:52	8:02	5:51	5:01

### MRI preprocessing

T1WI were acquired on 3.0-Tesla scanners. All processing was performed using MATLAB (Mathworks, Inc., Natick, MA, USA), SPM12 (Statistical Parametric Mapping software package version 12, https://www.fil.ion.ucl.ac.uk/spm/software/), and CAT (A Computational Anatomy Toolbox for SPM) 12 toolbox (http://www.neuro.uni-jena.de/cat/). The CAT12 toolbox provided the DARTEL algorithm 19 and a template of healthy subjects in DARTEL by the IXI-database (http://brain-development.org/). In preprocessing, T1WI was segmented for gray matter (GM) and white matter (WM) by segmentation in SPM. Next, diffeomorphic anatomical registration was performed on the GM and WM images to construct a template for coregistration for each participant [19]. All imaging data were registered to the MNI template space. Finally, these images were smoothed 8 mm full-width at half-maximum by a Gaussian kernel [20] and transformed into Montreal Neurological Institute stereotactic space using affine and nonlinear spatial normalization.

### ComBat harmonization

ComBat harmonization is a batch-effect correction tool introduced for use in genetic research using microarrays [21]. This tool was shown to remove intersite technical variability while preserving intersite biological variability in multi-site neuroimaging studies [16, 22, 23]. In this study, we applied ComBat harmonization to each voxel in the T1WI data for each subject with eating disorder diagnosis, age, intracranial volume, and body mass index (BMI) as biological covariates. Therefore, all data were corrected only for site variability.

### Statistical analysis

#### Demographic and clinical characteristics

Demographic variables were analyzed and compared using SPSS (version 27.0; SPSS Inc., Chicago, IL, USA). Age, BMI, total brain volume (TBV), and EDE-Q scores were compared between groups using Student’s *t* test. In all analyses, *P* < 0.05 was taken to indicate statistical significance.

#### Voxel-based morphometry (VBM)

For GMV data, the two-sample *t* test was computed in SPM using VBM to compare AN and HC groups, as well as ANR vs. HC, ANBP vs. HC, and ANR vs. ANBP (See eMethod). Age and TBV were used as nuisance covariates. Particularly, TBV was applied to correct for differences in brain size between participants. All images were thresholded with an absolute threshold of < 0.1. Group comparisons used a height threshold of *P* < 0.001, and clusters were considered statistically significant at *P* < 0.05 by family-wise error (FWE) at the cluster level for multiple comparisons. Correlation analyses were performed to assess relations between regional brain volume and EDE-Q scores in AN, ANR, ANBP, and HC groups in SPM using VBM with whole-brain analysis. Given the use of the four subscales of the EDE-Q questionnaire, we applied a correction for multiple comparisons in the correlation analyses. Specifically, we set the voxel level threshold at P < 0.001/4 for the four subscales, thus adjusting for the multiple testing. In the analysis using the global EDE-Q score, which does not involve multiple comparisons, we used a height threshold of *P* < 0.001. Extent threshold were considered statistically significant at *P* < 0.05 by FWE at the cluster level for multiple comparisons.

## Results

### Demographic and clinical characteristics

Table 3 shows the demographic and clinical characteristics of the study population. There was no significant difference in age between AN and HC groups, while BMI and TBV were significantly lower in the AN group than the HC group. EDE-Q scores were recorded for 87 participants in the AN group (51 ANR, 36 ANBP) and 92 HC. All EDE-Q scores were significantly higher in AN, ANR, and ANBP groups compared to the HC group. The ANBP subgroup showed significantly higher EDE-Q global score and subscores for Restraint, Eating Concern, and Shape Concern than the ANR subgroup, consistent with some previous studies of differences in clinical symptoms among AN subtypes [24–26].

**Table 3:** Demographic and clinical characteristics of all participants and participants with EDE-Q scores.

	All participants			Participants with EDE-Q score							
	AN (*n* = 103)	HC (*n* = 102)	AN vs. HC	AN (*n* = 87)	HC (*n* = 92)	AN vs. HC	ANR (*n* = 51)	ANBP (n = 36)	ANR vs. HC	ANBP vs. HC	ANR vs. ANBP
	mean (SD)	mean (SD)	*P*	mean (SD)	mean (SD)	*P*	mean (SD)	mean (SD)	*P*	*P*	*P*
Age (years)	33.11 (12.17)	31.31 (11.04)	0.271	34.07 (12.10)	31.37 (11.29)	0.124	30.29 (12.10)	39.42 (10.03)	0.596	<0.001	<0.001
BMI (kg/m^2^)	14.74 (2.25)	20.84 (2.59)	<0.001	14.48 (2.17)	20.82 (2.67)	<0.001	14.02 (2.14)	15.12 (2.08)	<0.001	<0.001	0.019
TBV (mL)	1039.96 (83.62)	1119.12 (85.02)	<0.001	1037.66 (85.87)	1120.07 (85.47)	<0.001	1037.57 (83.92)	1037.81 (89.75)	<0.001	<0.001	0.990
EDE-Q Restraint				2.16 (1.78)	0.48 (0.74)	<0.001	1.58 (1.55)	2.98 (1.76)	<0.001	<0.001	<0.001
EDE-Q Eating Concern				2.21 (1.80)	0.21 (0.47)	<0.001	1.71 (1.50)	2.92 (1.96)	<0.001	<0.001	0.002
EDE-Q Shape Concern				2.77 (1.71)	1.21 (1.11)	<0.001	2.27 (1.57)	3.48 (1.67)	<0.001	<0.001	0.001
EDE-Q Weight Concern				2.48 (1.74)	0.91 (0.95)	<0.001	2.07 (1.59)	3.07 (1.80)	<0.001	<0.001	0.008
EDE-Q global score				2.40 (1.60)	0.71 (0.72)	<0.001	1.91 (1.40)	3.11 (1.61)	<0.001	<0.001	<0.001

*AN* anorexia nervosa, *ANR* anorexia nervosa restricting type, *ANBP* anorexia nervosa binge-purging type, *HC* healthy controls, *BMI* body mass index, *TBV* total brain volume, *EDE-Q* Eating Disorder Examination Questionnaire 6.0, *SD* standard deviation.

### Comparison of VBM between groups

**Fig. 1:**
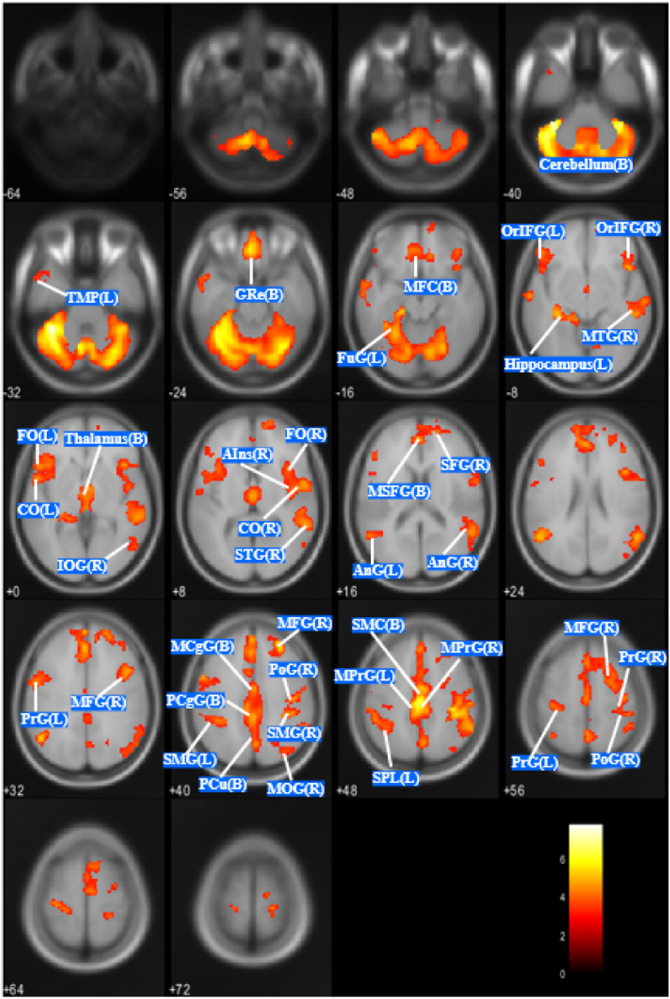
Regions of GMV reduction in AN compared with HC (corrected for age and TBV). *P* < 0.05 (corrected for multiple comparison family-wise error at the cluster level). TMP temporal pole, GRe gyrus rectus, MFC medial frontal cortex, FuG fugiform gyrus, OrIFG orbital part of the inferior frontal gyrus, MTG middle temporal gyrus, FO frontal operculum, CO central operculum, IOG inferior occipital gyrus, AIns anterior insula, STG superior temporal gyrus, SFG superior frontal gyrus, MSFG superior frontal gyrus medial segment, AnG angular gyrus, PrG precentral gyrus, MFG middle frontal gyrus, MCgG middle cingulate gyrus, PCgG posterior cingulate gyrus, PoG postcentral gyrus, SMG supramarginal gyrus, PCu precuneus, MOG middle occipital gyrus, SMC supplementary motor cortex, MPrG precentral gyrus medial segment, SPL superior parietal lobule. (B) bilateral; (L), left; (R), right.

In the analyses with age and TBV as nuisance covariates, the AN group showed significant decreases in GMV in widespread regions in the brain (Fig. 1). Cluster regions and peak level Montreal Neuroimaging Institute (MNI) coordinates are shown in eTable 1 in the Online-Only Materials. The AN group showed significantly reduced GMV in 10 clusters (clusters A–J), i.e., the cerebellum, middle and posterior cingulate gyrus, anterior and posterior insula, frontal lobe (supplementary motor cortex, medial/superior/middle/inferior frontal gyrus, gyrus rectus, precentral gyrus, and operculum), temporal lobe (superior/middle temporal gyrus, temporal pole, and fusiform), parietal lobe (angular gyrus, precuneus, postcentral gyrus, and superior parietal cortex), and thalamus.

### Correlation analysis of VBM

**Fig. 2:**
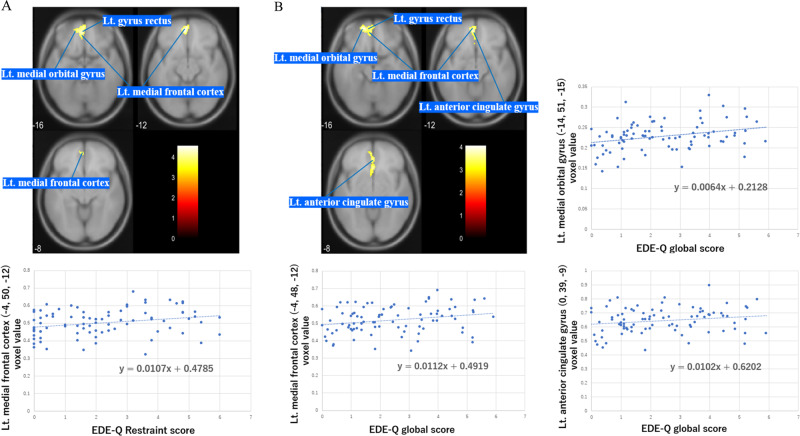
Ventromedial prefrontal regions showing a significant positive correlation with the severity of clinical symptoms. Region of GMV positively correlated with EDE-Q Restraint (**A**) and EDE-Q global score (**B**) in AN with a cluster threshold of FWE-corrected. *P* < 0.05 (corrected for age and TBV).

**Fig. 3:**
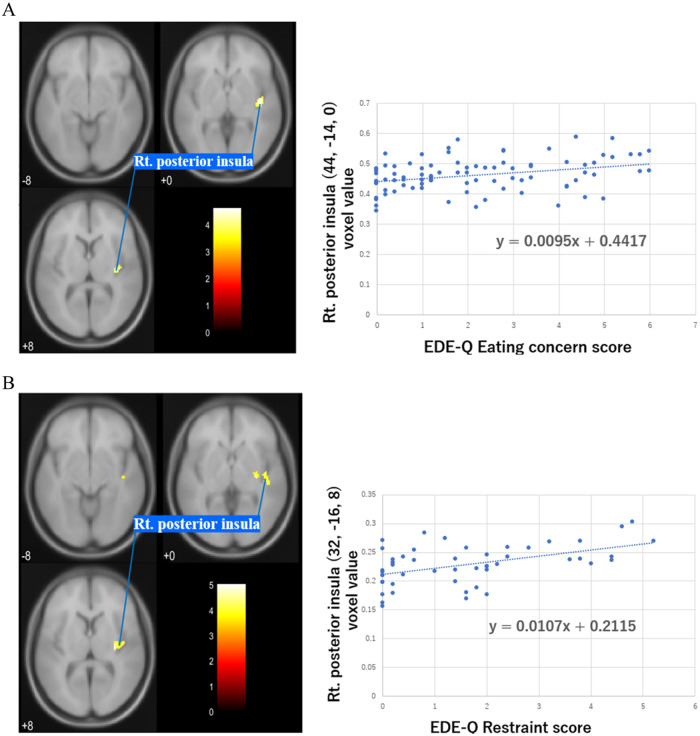
Posterior insula regions showing a significant positive correlation with the severity of clinical symptoms. Region of GMV positively correlated with EDE-Q Eating Concern in AN (**A**) and EDE-Q Restraint in ANR (**B**) with a cluster threshold of FWE-corrected. *P* < 0.05 (corrected for age and TBV).

In the AN group, the EDE-Q Restraint score was positively correlated with GMV in the large clusters including the left ventromedial frontal cortex (vmPFC) (x = −4, y = 50, z = −12; *P* < 0.004) (Table 4-1, Fig. 2-A). The EDE-Q global score was positively correlated with GMV in the large cluster including the left OFC (*x* = −14, *y* = 51, *z* = −15; *P* < 0.014), left vmPFC (*x* = −4, *y* = 48, *z* = −12; *P* < 0.014), and left rostral anterior cingulate gyrus (rostral ACC) (*x* = 0, *y* = 39, *z* = −9; *P* < 0.014) (Table 4-2, Fig. 2B). The EDE-Q Eating Concern scores were positively correlated with GMV in the right posterior insula (*x* = 44, *y* = −14, *z* = 0; *P* < 0.038) (Table 4-3, Fig. 3-A). In the ANR subgroup, the EDE-Q Restraint scores were positively correlated with GMV in the large clusters including the right posterior insula (*x* = 32, *y* = −16, *z* = 8; *P* < 0.003) (Table 4-4, Fig. 3-B).

**Table 4:** The results of Correlation analyses.

4-1: Regions of GMV Positively Correlated With EDE-Q Restraint in AN (Corrected for Age and TBV)						
Region	*P*	Cluster size	Peak level MNI coordinates			*t*-value
			*x*	*y*	*z*	
Lt. medial frontal cortex	0.004*	598	−4	50	−12	4.55

4-2: Regions of GMV Positively Correlated With EDE-Q Global Score in AN (Corrected for Age and TBV)						
Region	*P*	Cluster size	Peak level MNI coordinates			*t*-value
			*x*	*y*	*z*	
Lt. medial orbital gyrus	0.014*	1071	−14	51	−15	4.05
Lt. medial frontal cortex			−4	48	−12	3.88
Lt. anterior cingulate gyrus			0	39	−9	3.70

4-3: Regions of GMV Positively Correlated With EDE-Q Eating Concern Score in AN (Corrected for Age and TBV)						
Region	*P*	Cluster size	Peak level MNI coordinates			*t*-value
			*x*	*y*	*z*	
Rt. posterior insula	0.038*	388	44	−14	0	4.62

4-4: Regions of GMV Positively Correlated With EDE-Q Restraint Score in ANR (Corrected for Age and TBV)						
Region	*P*	Cluster size	Peak level MNI coordinates			*t*-value
			*x*	*y*	*z*	
Rt. posterior insula	0.003*	599	32	−16	8	5.04

*P < 0.05 family-wise error-corrected at cluster level.

*AN* anorexia nervosa, *ANR* anorexia nervosa restricting type, *EDE-Q* Eating Disorder Examination Questionnaire 6.0, *GMV* gray matter volume, *MNI* Montreal Neuroimaging Institute, *TBV* total brain volume.

There were no significant correlations between GMV and EDE-Q scores in ANBP and HC groups.

## Discussion

In this study, analysis of the MRI data for the whole brain rather than ROIs in over 100 participants with AN allowed us to obtain reliable results. Global areas of GMV reduction were observed in AN, i.e., GMV reductions were seen in the bilateral cerebellum, middle and posterior cingulate gyrus, supplementary motor cortex, precentral gyrus medial segment, and thalamus, which were mostly consistent with the results of a previous voxel-wise meta-analysis of AN 13. In addition, the OFC, vmPFC, rostral ACC, and posterior insula volumes were positively correlated with the severity of AN symptoms.

The morphological changes observed in AN were consistent with previous reports and consolidated conventional understanding of the pathogenesis of AN. A recent voxel-wise meta-analysis of AN reported volume reduction in the bilateral cerebellum, bilateral middle and posterior cingulate gyrus, bilateral precuneus, and bilateral supplementary motor cortex [13]. Given the consistency with our findings of whole-brain MRI analysis in a large population of patients with AN, the observed volume reductions in these brain areas in AN were robust and reliable. Interpretations of volume reduction in each region are discussed in eDiscussion of the Online-Only Materials.

While additional factors such as BMI, malnutrition, gonadal hormones, and genetic predispositions may influence gray matter volume in anorexia nervosa, our findings, which correct for total brain volume, suggest that the observed regional reductions reflect the specific pathophysiology of the disorder. There are several other factors potentially influencing the observed regional GMV reductions in AN, beyond the presence of comorbid conditions such as anxiety and depression [27, 28]. Notably, while we included age as a covariate in our analysis to control for its effect, other variables such as BMI and malnutrition could not be entirely ruled out. Previous research has suggested a strong correlation between BMI and TBV, which could indicate that malnutrition or low BMI significantly contributes to overall reductions in brain volume 12. Nonetheless, our analysis, which corrected for TBV, showed regional GMV reductions that we believe reflect the specific pathophysiology of AN. Additionally, while data on gonadal hormones were not available in this study, their potential influence on brain volume cannot be entirely excluded. In AN patients, changes in gonadal hormone levels, particularly relating to amenorrhea driven by low body weight, could have a substantial impact on global rather than local GMV reductions. Also, genetic predispositions related to AN might play a role. However, the overall genetic contribution to AN is not as substantial as might be expected. As suggested by a genome-wide association study [29], the odds ratio for genetic contribution was found to be at most 1.17. Therefore, although genetic factors likely contribute to the onset and course of AN, they may not be the most crucial determinants. Taken together, while these additional factors certainly warrant further investigation, our current findings provide valuable insights into the brain alterations associated with AN.

The relatively large GMV in the vmPFC and OFC were associated with the severity and food restraint of AN, suggesting enhancement of two top-down cognitive control processing pathways: i.e., cognitive control of reward processing and emotional processing. It has been reported that abnormalities in the reward and emotional processing systems are closely involved in the pathogenesis of AN. The OFC and vmPFC have top-down control functions to the ventral striatum, and these regions along with the rostral ACC exert inhibitory top-down control over the amygdala, which is the main brain region involved in processing negative emotions [30]. More precisely, the GMV of the bilateral gyrus rectus (GRe) and medial frontal cortex (MFC), adjacent to the orbitofrontal cortex (OFC), was observed to be reduced in AN patients compared to HC. This reduction suggests a generalized impairment associated with the GRe and MFC in patients with AN. On the other hand, no such GMV reduction was noted in the left medial orbital gyrus and left anterior cingulate gyrus. Rather, these latter regions may show a pathological enhancement in function, particularly as the severity of dietary restriction symptoms increases in AN patients. Given our results of correlation analyses, the association between enhanced reward and emotional control processing and AN symptoms can be considered to involve two pathways.

First, the positive correlations between EDE-Q scores (i.e., global score and Restraint subscale) and regional GMV in the OFC and vmPFC suggested that the extreme food restriction induced by long-term top-down control of reward processing may be associated with the relative enlargement in these areas. The vmPFC and the OFC have top-down control functions on the ventral striatum, which plays an important role in the reward system [31]. An aberrant reward processing system is a key potential mechanism of AN, manifestations of which are seen in the OFC [32, 33]. The OFC, an important higher-order brain region for processing of reward expectation and value [34], aids in controlling how much we eat [35], and has been reported to be associated with food avoidance [36]. Previous studies reported increased GMV [37] and cortical thickness [8] of the OFC in patients with AN. The relatively larger OFC volume within individuals with AN is thought to be associated with increased reward and punishment sensitivity to eating in eating disorders, leading to cognitive control over eating and avoidance of food [37]. The findings of the present study were consistent with this previous report highlighting the function of the OFC as a top-down mechanism of suppressing food cravings and, therefore, the volume of the OFC may be related to the severity of eating disorders.

Second, the positive correlations between EDE-Q scores (i.e., global score and Restraint subscale) and regional GMV in the OFC, vmPFC, and rostral ACC suggested that the extreme food restriction induced by long-term top-down control of emotional processing may also be associated with relative enlargement in these areas. The vmPFC has been implicated in the processing of risk and fear regulating amygdala activity in humans [38]. In addition, research on phobic conditioning has shown that the activity of the amygdala decreases as the activity of the OFC and vmPFC increases along with the rostral ACC with loss of fear learning. In addition, the ACC is a multimodal region involved in affective processing [39], and the rostral ACC especially modulates fear processing [40]. The rostral ACC is strongly interconnected with the OFC and amygdala, and is involved in higher-order functions, such as conditioned emotional learning, assessment of motivational content, and assigning emotional valence to internal and external stimuli [41], and is primarily involved in assessing the salience of emotional and motivational information and regulation of emotional responses [39]. Taken together, these observations indicate that these regions exert inhibitory top-down control over the amygdala [42]. Activation of the vmPFC is associated with successful suppression of emotional responses to negative emotional signals [43]. In fact, women with eating disorders showed greater activation in the vmPFC and OFC in response to food stimuli that they identified as threatening and disgusting [44]. Therefore, the positive correlations between the volumes of these regions and dietary restriction in AN patients may be explained by top-down control suppressing food cravings and other emotions.

Relative enlargement of GMV in the posterior insula was associated with Eating Concern and Food Restraint subscales in AN and ANR only, respectively, indicating that these AN symptoms were induced by enhanced awareness of somatic signal processing. The insula is involved in integrating homeostatic and emotional information, with connections to the limbic and cortical areas [45]. The posterior part of the insula processes interoceptive information in the body [46–48], and is involved in processing somatosensory stimuli with affective or motivational significance [49]. As the stage of processing progresses from the posterior insula to the anterior insula, interoceptive information is processed in an integrated manner with cognitive and emotional information. One study reported that GMV of the posterior insula was positively correlated with the duration of illness and dissatisfaction with body shape in AN [50]. In patients with AN, the volume of the posterior insula is thought to be relatively large due to stimulation of the gastrointestinal tract by hunger from a young age [50], and the pathway from the posterior insula to behavior-determining regions, such as the OFC, is enhanced, which may be involved in behaviors, such as extreme dietary restriction. In the present study, the GMV in the right posterior insular cortex was significantly decreased in AN patients compared to HC, while the GMV in the left posterior insular cortex was not significantly decreased. These results suggest that right posterior insular cortex function was generally reduced in AN patients. In contrast, the left posterior insular cortex function could be associated with enhanced somatic signal processing, which appeared to symptoms of eating concern and food restriction progressed. Although functional differences between the left and right posterior insular cortex have not been revealed in previous neuroimaging studies in AN, the present results suggest that the processing of somatic signals involving the left posterior insular cortex can be more involved in the progression of pathology in AN. Through the mechanisms described above, dietary restriction and obsession with food may be associated with the relative enlargement of the posterior insular volume within individuals with AN. That is, the eating concern in AN and excessive food restriction in ANR may be induced by somatic signal processing from the body. Specifically, AN patients may overprocess body signals related to their own body shape and appearance, and they may therefore be concerned about the ways in which people look at them, described as an EDE-Q item. It is also conceivable that excessive food restriction in ANR may be induced by the frequent processing of body signals, such as stomach pain and nausea, to suppress eating behavior.

In our discussion, we proposed that the relatively large GMV observed in the vmPFC and OFC may indicate an enhancement of top-down cognitive control. Similarly, the findings in the posterior insula may suggest an enhancement of the awareness of somatic signal processing. This interpretation was informed by existing literature, such as Kanai et al., 2011, which often infers functional implications from structural alterations [51]. However, we recognize that these interpretations should be made with caution, as they cannot confirm causality. Therefore, while we believe our findings contribute to the understanding of potential brain mechanisms in AN, we acknowledge that our discussion on this matter may have been speculative. Future studies employing both structural and functional measures are necessary to verify these proposed mechanisms.

This study had some limitations. This study was conducted by means of a multicenter collaboration to obtain a large sample size, which was its greatest strength, but the sample sizes from some centers were very small. These differences in sample size between centers may have had a relatively large influence on the results. In addition, the duration of illness was not taken into account, so it was not possible to determine whether the changes identified in this study appear early or develop later in the course of the disease. Eating disorders are often associated with comorbidities [52]. Generally, the comorbidities in AN are thought to include depression and anxiety, which often appear secondary to AN [53, 54], but our data did not include detailed information about comorbidities. Our study was further constrained by the absence of comprehensive clinical data on psychiatric comorbidities such as depression and anxiety. While our additional analyses found no significant correlation between these conditions and observed gray matter volume reductions (see supplementary results), suggesting that the GMV reductions we observed in the AN group are more likely associated with the condition of AN itself rather than comorbid depression or anxiety. Future research with more extensive psychiatric profiles would bolster our understanding of the effects of these comorbidities on AN.

Further, longitudinal studies are also needed to follow the subsequent course of severe and chronic cases and to determine changes over time before and after treatment in order to determine how the brain regions identified in this study relate to the pathophysiology of AN and its response to treatment. In fact, our multicenter research team has initiated a longitudinal study before and after treatment for eating disorders [55].

## Conclusion

The present study was conducted with data from a large number of participants in a multicenter collaboration, to identify brain morphological abnormalities and disease-specific neurological biomarkers of AN. Consistent with the results of previous studies, the GMV of the bilateral cerebellum, bilateral middle and posterior cingulate gyrus, and bilateral supplementary motor cortex were reduced in AN regardless of subtype, and these alterations may explain the core pathology of AN. Furthermore, whole-brain analysis showed positive correlations of severity with GMV of regions involved in the reward system and emotion regulation, such as the OFC, rostral ACC, and vmPFC. These regions elicit top-down control in AN, and may be related to the severity of AN symptoms. GMV of the posterior insula, which processes interoceptive information in the body, was also positively correlated with severity. These regions may be useful as biomarkers of the severity of AN.

### Supplementary information

Supplementary information

## Data Availability

Data from participants who agreed to the public distribution of data are available from the corresponding author upon reasonable request.
