# Supplementary material for: Systematic reduction of gray matter volume in anorexia nervosa, but relative enlargement with clinical symptoms in the prefrontal and posterior insular cortices: a multicenter neuroimaging study
Source: Mol Psychiatry. 2024 Jan 22;29(4):891–901. doi: 10.1038/s41380-023-02378-4 (PMC11176065; doi:10.1038/s41380-023-02378-4)
Supplement: Supplementary file 1 — Supplementary information [file 41380_2023_2378_MOESM1_ESM.docx]

**Online-Only Materials**

**eMethods**

**eResults**

**eDiscussion**

**eReferences**

**eTable 1.** Region of Decreased GMV in AN Compared With HC

**eTable 2.** Demographic and Clinical Characteristics of Participants With BDI and STAI scores

**eTable 3.** Demographic and Clinical Characteristics of Participants With ANR and ANBP

**eTable 4.** Region of Decreased GMV in ANR Compared With HC

**eTable 5.** Region of Decreased GMV in ANBP Compared With HC

**eTable 6.** Regions Reported in Previous Studies Decreased GMV and Cortical Thickness in AN Compared With HC

**eTable 7.** Regions that Showed Positive Correlation in the Correlation Analysis and the Group Differences in the Current Study and Reports of Previous Studies in those Regions

**eFigure 1.** Regions of GMV Reductions in ANR Compared With HC

**eFigure 2.** Regions of GMV Reductions in ANBP Compared With HC

**eMethods**

## **Additional psychological datasets**

Of the 205 participants, the state-trait anxiety scores were available for 195 participants, consisting of 94 with AN and 101 HCs using STAI (State-trait anxiety inventory) 56 57. Also, the depressive symptom scores were available for 180 participants, consisting of 85 with AN and 95 HCs using BDI-II (Beck depression inventory-II) 58 59.

**Demographic and clinical characteristics**

All of STAI (State-trait anxiety inventory) and BDI-II (Beck depression inventory-II) scores were significantly higher in the AN group than in HC (eTable 2).

eTable 3 shows the demographic and clinical characteristics of participants. There was no significant difference in age between ANR and HC, but BMI and TBV were significantly lower in ANR. The ANBP subgroup was significantly older and had lower BMI and TBV than HCs. The ANBP subgroup was significantly older and had higher BMI than the ANR subgroup, but there was no significant difference in TBV.

**Statistical analyses on voxel-based morphometry**

We also compared ANR vs. HC, ANBP vs. HC, and ANR vs. ANBP. Demographic variables were analyzed and compared using SPSS. Age, BMI, and TBV were compared between groups using Student’s *t* test. Group comparisons used a height threshold of *P* < 0.001, and clusters were considered statistically significant at *P* < 0.05 by FWE at the cluster level for multiple comparisons. For GMV data, the two-sample *t* test was computed in SPM using voxel-based morphometry (VBM) to compare ANR vs. HC, ANBP vs. HC, and ANR vs. ANBP. In the analyses, age and TBV were applied as nuisance covariates.

**eResults**

**Comparison between groups in VBM**

No significant clusters of increased GMV were found in ANR and ANBP compared to HC. ANR showed significantly reduced GMV in eight clusters (clusters K–R) (eTable 4, eFigure 1), and these regions were similar to those where volume loss was observed in AN. ANBP showed significantly reduced GMV in seven clusters (clusters S–Y) (eTable 5, eFigure 2). Thus, ANBP showed fewer areas of volume loss than AN and ANR. There was no significant difference in GMV between ANR and ANBP groups.

**eDiscussion**

The GMV reduction in the middle to posterior cingulate gyrus reflects stubborn dietary restriction in AN, caused by reduced flexibility in thinking via dysfunction of the default mode network 13. The middle cingulate gyrus is involved in the processing of emotions, and the anterior subregion of this area is thought to be involved in fear and avoidance behavior 60. Degeneration of the middle cingulate gyrus may partly explain the symptoms of AN, such as excessive inhibition, anxiety, depression, and alexithymia 13. The posterior cingulate gyrus and precuneus are involved in the default mode network (DMN), and reduced DMN activity in AN has also been consistently reported in functional MRI studies 61^,^ 62. Therefore, the middle and posterior cingulate gyrus is a candidate brain region involved in dietary restriction in AN via DMN function and related cognitive functions.

Reduced GMV in the cerebellum and sensorimotor network (SMN), including the supplementary motor area and thalamus, showed associations with core cognitive symptoms of AN, such as cognitive distortion of body image and cognitive behavioral inflexibility. Some studies showed that the cerebellum is involved in the regulation of various visceral functions, including feeding control, and that cerebellar pathological changes may induce alterations in feeding behavior and consequent decrease in body weight 63. It has been suggested that cerebellar volume alterations may contribute to dietary restriction in AN 13. In addition, a functional and structural MRI study showed reduced SMN activity and volume in AN 62^,^ 64. SMN impairment in AN reflects degenerated processing of somatosensory information regarding body size perception 65 as well as cognitive behavioral inflexibility 66^,^ 67. Therefore, degeneration of the cerebellum and SMN may explain cognitive distortion of body image, cognitive behavioral inflexibility, and dietary restriction in AN.

The results of the present study did not reveal any differences in brain structures between ANR and ANBP groups. In contrast, one previous study reported reduced volume of the left OFC, right anterior insula, bilateral parahippocampal gyrus, and left cerebellum in ANR compared to ANBP 68 among 14 individuals with AN. This discrepancy may have been partly due to differences in sample size between the studies. In addition, it may be difficult to detect significant differences on brain imaging because subjects with ANR can develop ANBP over time 69, which is known as diagnostic migration in eating disorder 70. To detect differences among subtypes, further studies with larger sample sizes of subjects with ANR only who do not develop ANBP, and those with ANBP, are required.

Finally, to provide a more comprehensive understanding of our findings within the context of existing literature, we have conducted a narrative review. This review carefully examined whether the brain regions we identified as showing GMV reductions in AN and significant correlations with EDE scales have been previously highlighted in the literature. We found that many of these regions correspond with brain areas indicated in individual studies with smaller sample sizes. For the benefit of the reader, we have tabulated the results of this narrative review and included it as eTable 6, 7 This table juxtaposes our findings with those from previous studies, enabling an easy comparison and helping to elucidate what our study replicates and what it uniquely contributes to the field.

# eReferences

56. Spielberger C, Gorsuch R, Lushene R, Vagg P, Jacobs G. Manual for the state-trait anxiety inventory. Consulting Psychologists Press: Palo Alto, CA, 1983.

57. Nakazato K, Mizuguchi T. How to use STAI. Sankyoubou Corp.: Kyoto, 1982.

58. Beck A, Steer R, G. B. BDI-II: Beck Depression Inventory. Second Edition. . The Psychological Corporation,: San Antonio, TX, 1996.

59. Kojima M, Furukawa TA, Takahashi H, Kawai M, Nagaya T, Tokudome S. Cross-cultural validation of the Beck Depression Inventory-II in Japan. Psychiatry research 2002; 110(3): 291-299.

60. Vogt BA, Finch DM, Olson CR. Functional heterogeneity in cingulate cortex: the anterior executive and posterior evaluative regions. Cereb Cortex 1992; 2(6): 435-443.

61. Sachdev P, Mondraty N, Wen W, Gulliford K. Brains of anorexia nervosa patients process self-images differently from non-self-images: an fMRI study. Neuropsychologia 2008; 46(8): 2161-2168.

62. McFadden KL, Tregellas JR, Shott ME, Frank GK. Reduced salience and default mode network activity in women with anorexia nervosa. J Psychiatry Neurosci 2014; 39(3): 178-188.

63. Zhu JN, Wang JJ. The cerebellum in feeding control: possible function and mechanism. Cellular and molecular neurobiology 2008; 28(4): 469-478.

64. Titova OE, Hjorth OC, Schiöth HB, Brooks SJ. Anorexia nervosa is linked to reduced brain structure in reward and somatosensory regions: a meta-analysis of VBM studies. BMC psychiatry 2013; 13: 110.

65. Favaro A, Santonastaso P, Manara R, Bosello R, Bommarito G, Tenconi E et al. Disruption of visuospatial and somatosensory functional connectivity in anorexia nervosa. Biological psychiatry 2012; 72(10): 864-870.

66. Zastrow A, Kaiser S, Stippich C, Walther S, Herzog W, Tchanturia K et al. Neural correlates of impaired cognitive-behavioral flexibility in anorexia nervosa. The American journal of psychiatry 2009; 166(5): 608-616.

67. Friederich HC, Herzog W. Cognitive-behavioral flexibility in anorexia nervosa. Curr Top Behav Neurosci 2011; 6: 111-123.

68. Brooks SJ, Barker GJ, O'Daly OG, Brammer M, Williams SC, Benedict C et al. Restraint of appetite and reduced regional brain volumes in anorexia nervosa: a voxel-based morphometric study. BMC psychiatry 2011; 11: 179.

69. Burton AL, Abbott MJ. Processes and pathways to binge eating: development of an integrated cognitive and behavioural model of binge eating. Journal of eating disorders 2019; 7: 18.

70. Milos G, Spindler A, Schnyder U, Fairburn CG. Instability of eating disorder diagnoses: prospective study. The British journal of psychiatry : the journal of mental science 2005; 187: 573-578.

71. Joos A, Kloppel S, Hartmann A, Glauche V, Tuscher O, Perlov E et al. Voxel-based morphometry in eating disorders: correlation of psychopathology with grey matter volume. Psychiatry research 2010; 182(2): 146-151.

72. Phillipou A, Rossell SL, Gurvich C, Castle DJ, Abel LA, Nibbs RG et al. Differences in regional grey matter volumes in currently ill patients with anorexia nervosa. The European journal of neuroscience 2018; 47(2): 177-183.

73. Boghi A, Sterpone S, Sales S, D'Agata F, Bradac GB, Zullo G et al. In vivo evidence of global and focal brain alterations in anorexia nervosa. Psychiatry research 2011; 192(3): 154-159.

74. Friederich HC, Walther S, Bendszus M, Biller A, Thomann P, Zeigermann S et al. Grey matter abnormalities within cortico-limbic-striatal circuits in acute and weight-restored anorexia nervosa patients. NeuroImage 2012; 59(2): 1106-1113.

75. Leppanen J, Sedgewick F, Cardi V, Treasure J, Tchanturia K. Cortical morphometry in anorexia nervosa: An out-of-sample replication study. European eating disorders review : the journal of the Eating Disorders Association 2019; 27(5): 507-520.

76. Nickel K, Joos A, Tebartz van Elst L, Matthis J, Holovics L, Endres D et al. Recovery of cortical volume and thickness after remission from acute anorexia nervosa. The International journal of eating disorders 2018; 51(9): 1056-1069.

77. Favaro A, Tenconi E, Degortes D, Manara R, Santonastaso P. Gyrification brain abnormalities as predictors of outcome in anorexia nervosa. Human brain mapping 2015; 36(12): 5113-5122.

78. Kohmura K, Adachi Y, Tanaka S, Katayama H, Imaeda M, Kawano N et al. Regional decrease in gray matter volume is related to body dissatisfaction in anorexia nervosa. Psychiatry Res Neuroimaging 2017; 267: 51-58.

79. Bomba M, Riva A, Morzenti S, Grimaldi M, Neri F, Nacinovich R. Global and regional brain volumes normalization in weight-recovered adolescents with anorexia nervosa: preliminary findings of a longitudinal voxel-based morphometry study. Neuropsychiatric disease and treatment 2015; 11: 637-645.

80. Castro-Fornieles J, Bargallo N, Lazaro L, Andres S, Falcon C, Plana MT et al. A cross-sectional and follow-up voxel-based morphometric MRI study in adolescent anorexia nervosa. Journal of psychiatric research 2009; 43(3): 331-340.

81. Gaudio S, Nocchi F, Franchin T, Genovese E, Cannata V, Longo D et al. Gray matter decrease distribution in the early stages of Anorexia Nervosa restrictive type in adolescents. Psychiatry research 2011; 191(1): 24-30.

82. Martin Monzon B, Henderson LA, Madden S, Macefield VG, Touyz S, Kohn MR et al. Grey matter volume in adolescents with anorexia nervosa and associated eating disorder symptoms. The European journal of neuroscience 2017; 46(7): 2297-2307.

83. Frank GK, Shott ME, Hagman JO, Yang TT. Localized brain volume and white matter integrity alterations in adolescent anorexia nervosa. J Am Acad Child Adolesc Psychiatry 2013; 52(10): 1066-1075 e1065.

## **eTable 1.** **Region of Decreased GMV in AN Compared With HC (Corrected for Age and TBV)**

| Cluster | Region | *P* | Cluster size |  | | Peak level | |  | | *t*-value | |
| --- | --- | --- | --- | --- | --- | --- | --- | --- | --- | --- | --- |
|  |  |  |  |  | MNI coordinates | | | |  | |  |
|  |  |  |  | x | | | y | z | |  | |
| A | Lt. cerebellum exterior | < 0.001 | 38119 | −38 | | | −50 | −30 | | 7.82 | |
| B | Lt. middle cingulate gyrus | < 0.001 | 28656 | −2 | | | −23 | 47 | | 6.43 | |
| C | Lt. gyrus rectus | 0.001 | 2058 | −3 | | | 39 | −22 | | 5.82 | |
| D | Lt. angular gyrus | 0.012 | 1307 | −50 | | | −60 | 28 | | 5.37 | |
| E | Rt. central operculum | < 0.001 | 4995 | 46 | | | −4 | 3 | | 5.31 | |
| F | Lt. middle temporal gyrus | 0.005 | 1615 | −62 | | | −10 | −10 | | 5.09 | |
| G | Lt. precentral gyrus | < 0.001 | 5321 | −51 | | | 4 | 39 | | 4.90 | |
| H | Lt. precentral gyrus | 0.026 | 1058 | −36 | | | −24 | 60 | | 4.88 | |
| I | Lt. thalamus proper | 0.003 | 1741 | 0 | | | −14 | 8 | | 4.73 | |
| J | Lt. superior parietal lobule | 0.007 | 1468 | −32 | | | −40 | 45 | | 4.69 | |

**P* < 0.05 family-wise error-corrected at cluster level.

AN, anorexia nervosa; GMV, gray matter volume; HC, healthy controls; MNI, Montreal Neuroimaging Institute; TBV, total brain volume.

| Cluster A: Bl. cerebellum exterior, Lt. fusiform gyrus, Rt. lingual gyrus. |
| --- |
| Cluster B: Bl. precentral gyrus medial segment, Bl. middle/posterior cingulate gyrus, Rt. superior/middle frontal gyrus, Bl. supplementary motor cortex, Bl. precuneus. |
| Cluster C: Bl. gyrus rectus, Bl. medial frontal cortex. |
| Cluster D: Lt. angular gyrus. |
| Cluster E: Rt. central/frontal operculum, Rt. anterior/posterior insula, Rt. orbital part of inferior frontal gyrus, Rt. posterior/lateral orbital gyrus, Rt. precentral gyrus. |
| Cluster F: Lt. middle/superior temporal gyrus, Lt. temporal pole. |
| Cluster G: Lt. precentral gyrus, Lt. middle frontal gyrus, Lt. frontal operculum, Lt. orbital/triangular part of the inferior frontal gyrus, Lt. anterior insula. |
| Cluster H: Lt. precentral/postcentral gyrus. |
| Cluster I: Bl. thalamus proper. |
| Cluster J: Lt. superior parietal lobule, Lt. supramarginal gyrus, Lt. angular gyrus. |

Bl., bilateral; Lt., left; Rt., right.

## **eTable 2. Demographic and Clinical Characteristics of Participants With BDI-II and STAI scores**

|  | Participants with BDI-II score | | |  |  | Participants with STAI score | | |
| --- | --- | --- | --- | --- | --- | --- | --- | --- |
|  | AN (n=94) | HC (n=101) | AN vs HC | |  | AN (n=85) | HC (n=95) | AN vs HC |
|  | mean (SD) | mean (SD) | *p* | |  | mean (SD) | mean (SD) | *p* |
| Age (years) | 33.65  (12.09) | 31.41  (11.05) | 0.177 | |  | 34.20  (12.17) | 30.89  (10.64) | 0.053 |
| BMI (kg/m²) | 14.57  (2.20) | 20.85  (2.60) | <0.001 | |  | 14.42  (2.20) | 20.83  (2.64) | <0.001 |
| BDI | 24.97  (13.20) | 6.32  (6.02) | <0.001 | |  |  |  |  |
| STAI  state |  |  |  | |  | 51.73  (12.93) | 36.83  (9.23) | <0.001 |
| STAI  trait |  |  |  | |  | 60.05  (15.48) | 42.89  (14.05) | <0.001 |

AN, anorexia nervosa; HC, healthy controls; BMI, body mass index; BDI, Beck Depression Inventory; STAI, State-Trait Anxiety Inventory; SD, standard deviation.

## **eTable 3.** **Demographic and Clinical Characteristics of ANR and ANBP Participants**

|  | ANR  (*n* = 58) | ANBP  (*n* = 45) | HC  (*n* =102) | ANR vs. HC | ANBP vs. HC | ANR vs. ANBP |
| --- | --- | --- | --- | --- | --- | --- |
|  | mean (SD) | mean (SD) | mean (SD) | *P* | *P* | *P* |
| Age (years) | 29.16 (12.02) | 38.2 (10.44) | 31.31 (11.04) | 0.251 | 0.001 | < 0.001 |
| BMI (kg/m^2^) | 14.30 (2.32) | 15.30 (2.05) | 20.84 (2.59) | < 0.001 | < 0.001 | 0.025 |
| TBV (mL) | 1040.77 (81.93) | 1038.90 (86.68) | 1119.12 (85.02) | < 0.001 | < 0.001 | 0.911 |

ANR, anorexia nervosa restricting type; ANBP, anorexia nervosa binge-purging type; BMI, body mass index; HC, healthy control; SD, standard deviation; TBV, total brain volume.

## **eTable 4. Regions of Decreased GMV in ANR Compared With HC (Corrected for Age and TBV)**

| Cluster | Region | *P* | Cluster size | |  | | Peak level | |  | | *t*-value |
| --- | --- | --- | --- | --- | --- | --- | --- | --- | --- | --- | --- |
|  |  |  |  |  | | MNI coordinates | | | |  |  |
|  |  |  |  | x | | | | y | z | |  |
| K | Lt. precentral gyrus medial segment | < 0.001 | 64741 | −2 | | | | −24 | 48 | | 7.03 |
| L | Rt. anterior insula | < 0.001 | 5940 | 44 | | | | 20 | −6 | | 6.36 |
| M | Rt. temporal pole | < 0.001 | 13393 | 41 | | | | 8 | −35 | | 6.21 |
| N | Lt. precentral gyrus | 0.001 | 2482 | −35 | | | | −24 | 60 | | 6.11 |
| O | Lt. angular gyrus | < 0.001 | 10133 | −27 | | | | −72 | 48 | | 5.40 |
| P | Lt. angular gyrus | 0.025 | 1150 | −50 | | | | −62 | 29 | | 5.26 |
| Q | Rt. thalamus | < 0.001 | 3606 | 2 | | | | −15 | 9 | | 4.77 |
| R | Lt. middle temporal gyrus | 0.001 | 2197 | −62 | | | | −10 | −10 | | 4.74 |

**P* < 0.05 family-wise error-corrected at cluster level.

AN, anorexia nervosa; GMV, gray matter volume; HC, healthy control; MNI, Montreal Neuroimaging Institute; TBV, total brain volume.

| Cluster K: Bl. cerebellum exterior, cerebellar vermal lobules, Lt. fusiform gyrus, Lt. lingual gyrus, Bl. precuneus, Bl. precentral gyrus medial segment, Bl. middle/posterior cingulate gyrus, Bl. supplementary motor cortex, Bl. superior frontal gyrus medial segment, Rt. superior/middle frontal gyrus, Bl. gyrus rectus, Bl. medial frontal cortex. |
| --- |
| Cluster L: Rt. anterior/posterior insula, Rt. frontal/central operculum, Rt. orbital part of the inferior frontal gyrus, Rt. planum polare, Rt. lateral orbital gyrus, Rt. triangular part of the inferior frontal gyrus. |
| Cluster M: Rt. temporal pole, Rt. precentral/postcentral gyrus, Rt. middle/superior temporal gyrus, Rt. angular gyrus, Rt. supramarginal gyrus, Rt. superior parietal lobule, Rt. planum temporale, Rt. parietal operculum, Rt. middle occipital gyrus. |
| Cluster N: Lt. precentral/postcentral gyrus, Lt. superior/middle frontal gyrus. |
| Cluster O: Lt. angular gyrus, Lt. superior parietal lobule, Lt. orbital/triangular part of inferior frontal gyrus, Lt. frontal/parietal operculum, Lt. supramarginal gyrus, Lt. precentral/postcentral gyrus, Lt. superior parietal lobule, Lt. anterior insula, Lt. planum temporale. |
| Cluster P: Lt. angular gyrus. |
| Cluster Q: Bl. thalamus proper, Rt. hippocampus. |
| Cluster R: Lt. middle/superior temporal gyrus, Lt. temporal pole. |

Bl., bilateral; Lt., left; Rt., right.

## **eTable 5. Regions of Decreased GMV in ANBP Compared With HC (Corrected for Age and TBV)**

| Cluster | Region | *P* | Cluster size |  | | Peak level | |  | | *t*-value |
| --- | --- | --- | --- | --- | --- | --- | --- | --- | --- | --- |
|  |  |  |  |  | MNI coordinates | | | |  |  |
|  |  |  |  | x | | | y | z | |  |
| S | Lt. cerebellum exterior | < 0.001 | 26296 | −38 | | | −48 | −28 | | 6.78 |
| T | Rt. middle frontal gyrus | 0.015 | 1259 | 46 | | | 12 | 32 | | 4.85 |
| U | Rt. middle cingulate gyrus | 0.006 | 1598 | 3 | | | −8 | 45 | | 4.81 |
| V | Lt. ventral diencephalon | 0.013 | 1317 | 0 | | | −3 | −10 | | 4.62 |
| W | Rt. angular gyrus | < 0.001 | 2899 | 53 | | | −57 | 24 | | 4.46 |
| X | Lt. precentral gyrus | 0.03 | 1039 | −53 | | | 0 | 33 | | 4.18 |
| Y | Lt. superior frontal gyrus medial segment | 0.033 | 1010 | −2 | | | 39 | 33 | | 4.05 |

**P* < 0.05 family-wise error-corrected at cluster level.

ANBP, anorexia nervosa binge-purging type; GMV, gray matter volume; HC, healthy controls; MNI, Montreal Neuroimaging Institute; TBV, total brain volume.

| Cluster S: Bl. cerebellum exterior, Bl. fusiform, Rt. lingual gyrus, Lt. hippocampus, Lt. parahippocampus. |
| --- |
| Cluster T: Rt. middle frontal gyrus, Rt. precentral gyrus, Rt. opercular part of the inferior frontal gyrus, Rt. central operculum, Rt. transverse temporal gyrus. |
| Cluster U: Bl. middle/posterior cingulate gyrus, Bl. supplementary motor cortex, Bl. precentral gyrus medial segment. |
| Cluster V: Bl. ventral diencephalon, Bl. thalamus proper. |
| Cluster W: Rt. angular gyrus, Rt. superior/middle temporal gyrus, Rt. supramarginal gyrus, Rt. postcentral gyrus, Rt. planum temporale, Rt. superior parietal lobule, Rt. middle occipital gyrus. |
| Cluster X: Lt. precentral gyrus, Lt. middle frontal gyrus, Lt. opercular part of the inferior frontal gyrus. |
| Cluster Y: Bl. superior frontal gyrus medial segment, Bl. anterior cingulate gyrus. |

Bl., bilateral; Lt., left; Rt., right.

## **eTable 6. Regions Reported in Previous Studies Decreased GMV and Cortical Thickness in AN Compared With HC**

| Cluster | Region with AN < HC | Previous studies |
| --- | --- | --- |
| A | Lt. cerebellum exterior | ④(v) ⑤(v) ⑪(v) |
| A | Rt. cerebellum exterior | ①(v) ②(v) ③(v) ⑤(v) ⑪(v) |
| A | Lt. fusiform gyrus | ④(v) ⑤(v) ⑪(v) |
| A | Rt. lingual gyrus | ⑨(c) ⑪(v) |
| B | Lt. precentral gyrus medial segment | ⑪(v) |
| B | Rt. precentral gyrus medial segment | ③(v) ⑤(v) ⑪(v) |
| B | Lt. middle cingulate gyrus | ⑨(c) ⑫(v) ⑬(v) ⑭(v) |
| B | Rt. middle cingulate gyrus | ⑨(c) ⑫(v) ⑬(v) ⑭(v) |
| B | Lt. posterior cingulate gyrus | ⑭(v) |
| B | Rt. posterior cingulate gyrus | ④(v) ⑭(v) |
| B | Rt. superior frontal gyrus | ①(c) ⑧(c) ⑨(c) ⑪(v) |
| B | Rt. middle frontal gyrus | ①(v) ③(v) ⑧(c) ⑪(v) |
| B | Lt. supplementary motor cortex | ⑪(v) |
| B | Rt. supplementary motor cortex | ⑤(v) ⑥(v) ⑪(v) ⑫(v) |
| B | Lt. precuneus | ①(c) ②(v) ⑫(v) ⑬(v) ⑭(v) |
| B | Rt. precuneus | ①(v,c) ⑦(c) ⑧(c) ⑨(c) ⑫(v) ⑬(v) ⑭(v) |
| C | Lt. gyrus rectus |  |
| C | Rt. gyrus rectus |  |
| C | Lt. medial frontal cortex | ⑭(v) |
| C | Rt. medial frontal cortex | ⑭(v) |
| D | Lt. angular gyrus | ①(v) ⑩(v) |
| E | Rt. central operculum |  |
| E | Rt. frontal operculum | ②(v) |
| E | Rt. anterior insula | ④(v) ⑥(v) ⑭(v) |
| E | Rt. posterior insula | ⑥(v) ⑭(v) |
| E | Rt. orbital part of inferior frontal gyrus |  |
| E | Rt. posterior orbital gyrus | ⑭(v) |
| E | Rt. lateral orbital gyrus | ⑭(v) |
| E | Rt. precentral gyrus | ⑤(v) |
| F | Lt. middle temporal gyrus | ①(v,c) ③(v) ⑤(v) ⑧(c) ⑩(v) ⑪(v) |
| F | Lt. superior temporal gyrus | ③(v) ⑪(v) |
| F | Lt. temporal pole | ⑥(v) |
| G | Lt. precentral gyrus | ⑪(v) |
| G | Lt. middle frontal gyrus | ⑨(v) ⑪(v) |
| G | Lt. frontal operculum |  |
| G | Lt. orbital part of the inferior frontal gyrus |  |
| G | Lt. triangular part of the inferior frontal gyrus | ⑧(c) |
| G | Lt. anterior insula | ⑥(v) ⑭(v) |
| H | Lt. precentral gyrus | ⑪(v) |
| H | Lt. postcentral gyrus | ⑪(v) ⑫(v) ⑬(v) |
| I | Bl. thalamus proper | ⑭(v) |
| J | Lt. superior parietal lobule | ⑦(c) ⑧(c) ⑪(v) ⑫(v) ⑬(v) |
| J | Lt. supramarginal gyrus | ⑪(v) ⑫(v) |
| J | Lt. angular gyrus | ①(v) ⑩(v) ⑪(v) |

AN, anorexia nervosa; HC, healthy controls; GMV, gray matter volume; (v), gray matter volume; (c), cortical thickness

|  | Studies | GMV/CT | Number of AN/HC |
| --- | --- | --- | --- |
|  | Mishima et al. (2021) 8 | GMV, CT | AN 35, HC 35 |
|  | Joos et al. (2010) 71 | GMV | AN 12, HC 18 |
|  | Phillipou et al. (2018) 72 | GMV | AN 26, HC 27 |
|  | Brooks et al. (2011) 68 | GMV | AN 14, HC 21 |
|  | Boghi et al. (2011) 73 | GMV | AN 21, HC 27 |
|  | Friederich et al. (2012) 74 | GMV | AN 12, HC 14 |
|  | Leppanen et al. (2019) 75 | CT | AN 46, HC 54 |
|  | Nickel et al. (2018) 76 | GMV, CT | AN 34, HC 41 |
|  | Favaro et al. (2015) 77 | CT | AN 38, HC 38 |
|  | Kohmura et al. (2017) 78 | GMV | AN 20, HC 29 |
|  | Bomba et al. (2015) 79 | GMV | AN 11, HC 8 (adolescents) |
|  | Castro-Fornieles et al. (2009) 80 | GMV | AN 12, HC 9 (adolescents) |
|  | Gaudio et al. (2011) 81 | GMV | AN 16, HC 16 (adolescents) |
|  | Martin Monzon et al. (2017) 82 | GMV | AN 26, HC 20 (adolescents) |

AN, anorexia nervosa; HC, healthy controls; GMV, grey matter volume; CT, cortical thickness

## **eTable 7. Regions that Showed Positive Correlation in the Correlation Analysis and the Group Differences in the Current Study and Reports of Previous Studies in those Regions**

| Region with positive correlation | Current study | Previous studies |
| --- | --- | --- |
| Lt. medial orbital gyrus |  | ①(c,↑) ⑭(v,↓) |
| Lt. orbitofrontal cortex (gyrus rectus) | (↓) | ⑮(v,↑)* ⑯(v,↑)* |
| Lt. medial frontal cortex | (↓) | ⑭(v,↓) |
| Lt. anterior cingulate gyrus |  | ②(v,↓) ⑥(v,↓) ⑦(c,↑) ⑩(v,↓) ⑭(v,↓) |
| Rt. posterior insula | (↓) | ⑭(v,↓) ⑰(v)* |

AN, anorexia nervosa; HC, healthy controls; GMV, gray matter volume; (v), gray matter volume; (c), cortical thickness; (↑), the volume in AN was greater than in HC; (↓), the volume in AN was lesser than in HC

⑮*: greater gyrus volume predicting a stronger pleasantness experience

⑯*: gyrus volume correlated negatively with sweet taste pleasantness

⑰*: greater gyrus volume were associated with longer duration of illness and body dissatisfaction

|  | Study | GMV/CT | The number of AN/HC |
| --- | --- | --- | --- |
| ① | Mishima et al. (2021) 8 | CT | AN 35, HC 35 |
| ② | Joos et al. (2010) 71 | GMV | AN 12, HC 18 |
| ⑥ | Friederich et al. (2012) 74 | GMV | AN 12, HC 14 |
| ⑦ | Leppanen et al. (2019) 75 | CT | AN 46, HC 54 |
| ⑪ | Kohmura et al. (2017) 78 | GMV | AN 20, HC 29 |
| ⑭ | Martin Monzon et al. (2017) 82 | GMV | AN 26, HC 20 |
| ⑮ | Frank et al. (2013) 37 | GMV | AN 19, HC 24 |
| ⑯ | Frank et al. (2013) 83 | GMV | AN 19, HC 22 |
| ⑰ | Zucker et al. (2017) 50 | GMV | AN 21, HC 20 |

AN, anorexia nervosa; HC, healthy controls; GMV, grey matter volume; CT, cortical thickness

## **eFigure 1. Regions of GMV Reduction in ANR Compared With HC (Corrected for Age and TBV; *P* < 0.05, Corrected for Multiple Comparison Family-wise Error at the Cluster Level)**


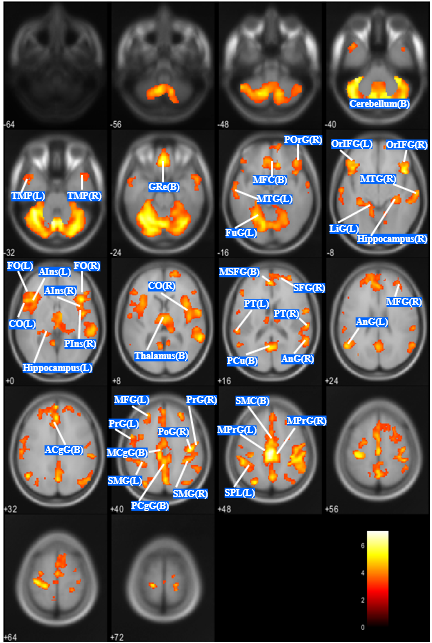


TMP, temporal pole; GRe, gyrus rectus; MFC, medial frontal cortex; MTG, middle temporal gyrus; FuG, fugiform gyrus; POrG, posterior orbital gyrus; OrIFG, orbital part of the inferior frontal gyrus; LiG, lingual gyrus; FO, frontal operculum; CO, central operculum; AIns, anterior insula; Pins, posterior insula; MSFG, superior frontal gyrus medial segment; SFG, superior frontal gyrus; PT, planum temporale; AnG, angular gyrus; PCu, precuneus; MFG, middle frontal gyrus; ACgG, anterior cingulate gyrus; MCgG, middle cingulate gyrus; PCgG, posterior cingulate gyrus; PrG, precentral gyrus; PoG, postcentral gyrus; SMG, supramarginal gyrus; SMC, supplementary motor cortex; MPrG, precentral gyrus medial segment; SPL, superior parietal lobule.

(B), bilateral; (L), left; (R), right.

## **eFigure 2. Regions of GMV Reduction in ANBP Compared With HC (Corrected for Age and TBV; *P* < 0.05, Corrected for Multiple Comparison Family-wise Error at the Cluster Level)**


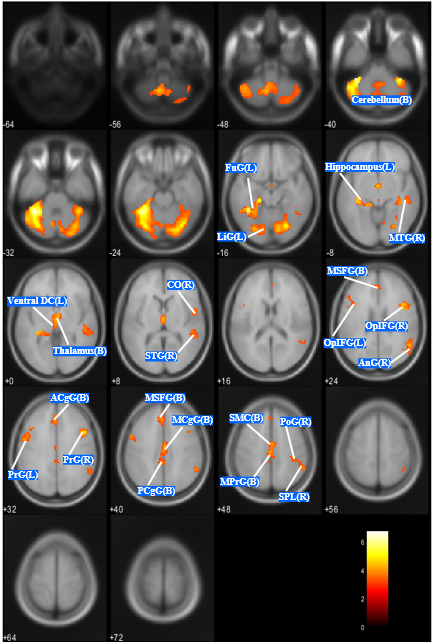


FuG, fugiform gyrus; LiG, lingual gyrus; MTG, middle temporal gyrus; CO, central operculum; STG, superior temporal gyrus; MSFG, superior frontal gyrus medial segment; OpIFG, opercular part of the inferior frontal gyrus; AnG, angular gyrus; ACgG, anterior cingulate gyrus; PrG, precentral gyrus; MCgG, middle cingulate gyrus; PCgG, posterior cingulate gyrus; SMC, supplementary motor cortex; MPrG, precentral gyrus medial segment; PoG, postcentral gyrus; SPL, superior parietal lobule.

(B), bilateral; (L), left; (R), right.
